# Our Public Institutions

**Published:** 1882-12

**Authors:** 


### Editorial.

---

### OUR PUBLIC INSTITUTIONS.

Following up the criticisms in which we have indulged, heretofore, of the public institutions of Buffalo, we are induced to refer to the excellent report of the medical officer, Dr. Stockton, of the Penitentiary, located in this city, in which he gives not only an account of his stewardship, but also offers some valuable suggestions in regard to the peculiar class that naturally gravitate to our penal institutions.

Several important recommendations are offered, which we endorse, inasmuch as they involve recognized principles in sanitary science. After referring in a complimentary manner to the ventilation, diet, cleanliness, hours of labor, etc., the report emphasizes the importance of "an isolation ward" for the proper care of "those afflicted with contagious diseases." The prevention of the spread of contagious diseases through isolation is one of the most essential measures now urged by sanitarians, and it cannot be questioned that in an institution to which is sent a class taken from the "slums" of a large city, suitable measures for separation should necessarily supplement the strict attention directed to personal cleanliness, diet, ventilation, etc. This measure should receive the prompt attention of the proper authorities.

We are glad to recognize the movement in favor of a resident medical attendant, and the value of a system of daily reports of cases, with the treatment adopted. This principle has been so far successful in other institutions, especially those devoted to the care of the insane, that the feasibility of its adoption cannot be questioned. The efforts of the State Board of Charities, to secure the appointment of a medical superintendent to our County Insane Asylum, are based upon the same principle and are substantiated by a like experience. The profession endorse the system of superintendence by educated officials of such institutions.

The necessity of more direct medical supervision than can be given through occasional visits by the non-resident medical officer, among so many prisoners as are confined in our Penitentiary, is very plain.

Upon the subject of short sentences for offenses, such as drunkenness, etc., the report says: "These people are seldom in a fit condition to labor when received, and many of them, during the entire period of their confinement, are carefully fed, reasonably well nursed, and also doctored, and have grown to look upon the institution not in the light of a workhouse or a penitentiary, but a kind of refuge place where they may resort for the process of "bracing up," that they may go out and sin and be sick again. Whether the present plan of sending such to this institution has in view either humanity or the public good, is a question not unworthy of the consideration of the proper persons. These people go out not sufficiently strengthened physically, not to say morally, to resist the influences that eventuate their return; and it would seem to me advisable that they be not sent here at all, or else that their term of confinement be sufficiently lengthened to do themselves and the county good."

This subject is a suggestive one, not so much to the profession as to the philanthropist, who desires to witness, as an outcome of existing punitive measures and agencies, some evidences of reformation or improvement in the lives and character of the misguided and the erring. The medical profession are too apt to estimate results obtained only through the application of principles of sanitary or medical science. The report incidently hints at the influence of physical strength over the moral sense; and it is too much to expect these victims of crime and dissipation to be improved, when neither time nor surrounding influences are allowed or granted for their physical and moral reformation.

As a whole, the report is the most suggestive and valuable one we have received from any of the public institutions of our city, and it is for this reason that we have taken occasion to refer to it at length. It is to be hoped that its recommendation will receive deserved attention from the Board of Supervisors. In the growth of our city in wealth and population, public officials must recognize the necessity of a broader liberality in dealing with its varied interests; and in the cause of humanity there is no excuse for parsimony in the provision offered for the amelioration of the condition of either the pauper or the criminal classes of the community.

